# Data recovery and integration from public databases uncovers transformation-specific transcriptional downregulation of cAMP-PKA pathway-encoding genes

**DOI:** 10.1186/1471-2105-10-S12-S1

**Published:** 2009-10-15

**Authors:** Chiara Balestrieri, Lilia Alberghina, Marco Vanoni, Ferdinando Chiaradonna

**Affiliations:** 1 Department of Biotechnology and Biosciences, University of Milano-Bicocca, Piazza della Scienza 2, 20126 Milano, Italy

## Abstract

**Background:**

The integration of data from multiple genome-wide assays is essential for understanding dynamic spatio-temporal interactions within cells. Such integration, which leads to a more complete view of cellular processes, offers the opportunity to rationalize better the high amount of "omics" data freely available in several public databases.

In particular, integration of microarray-derived transcriptome data with other high-throughput analyses (genomic and mutational analysis, promoter analysis) may allow us to unravel transcriptional regulatory networks under a variety of physio-pathological situations, such as the alteration in the cross-talk between signal transduction pathways in transformed cells.

**Results:**

Here we sequentially apply web-based and statistical tools to a case study: the role of oncogenic activation of different signal transduction pathways in the transcriptional regulation of genes encoding proteins involved in the cAMP-PKA pathway. To this end, we first re-analyzed available genome-wide expression data for genes encoding proteins of the downstream branch of the PKA pathway in normal tissues and human tumor cell lines. Then, in order to identify mutation-dependent transcriptional signatures, we classified cancer cells as a function of their mutational state. The results of such procedure were used as a starting point to analyze the structure of PKA pathway-encoding genes promoters, leading to identification of specific combinations of transcription factor binding sites, which are neatly consistent with available experimental data and help to clarify the relation between gene expression, transcriptional factors and oncogenes in our case study.

**Conclusions:**

Genome-wide, large-scale "omics" experimental technologies give different, complementary perspectives on the structure and regulatory properties of complex systems. Even the relatively simple, integrated workflow presented here offers opportunities not only for filtering data noise intrinsic in high throughput data, but also to progressively extract novel information that would have remained hidden otherwise. In fact we have been able to detect a strong transcriptional repression of genes encoding proteins of cAMP/PKA pathway in cancer cells of different genetic origins. The basic workflow presented herein may be easily extended by incorporating other tools and can be applied even by researchers with poor bioinformatics skills.

## Background

Integration achieves one of the most important imperatives of systems biology, namely it reduces the dimensionality of global data needed to deliver useful information about the networks active in the system of interest. The integration of data from different sources provides an effective means to deal with this issue by reinforcing *bona fide* observations and reducing false negatives. Moreover, because different experimental technologies provide different insights into a system, the integration of multiple data types offers the greatest information about a particular cellular process [1-3]. For example, gene perturbation experiments (e.g., knockouts or RNA interference) and microarrays analysis can reveal relationships between genes that may imply direct physical interactions or indirect logical interactions. Indeed, microarray experiments permit us to look at overall patterns of gene expression in order to understand the architecture of genetic regulatory networks, a global approach that could ultimately lead to complete description of the transcription-control mechanisms in a cell. In contrast, chromatin immunoprecipitation (ChIp) data can reveal direct protein-DNA interactions or cofactor associations with bound transcription factors. Combined together, these technologies can provide a much more detailed view of a transcriptional regulatory network than either alone.  

Several recent methods have addressed the problem of heterogeneous data integration and network prediction by modeling the noise inherent in high-throughput genomic datasets, especially by using statistical methods, which can significantly improve specificity and sensitivity and allow the robust integration of datasets with heterogeneous properties [4,5]. However, many of these methods recently developed to implement our ability to integrate and compare heterogeneous data, are often not easy to use and/or not freely accessible [6,7]. Taking into consideration that the development of efficient methods that facilitate the biological interpretation of these data is crucial, in the present work we focus on efficient identification of regulatory mechanisms, and propose an approach for analysis and interpretation of gene expression data based on the integration of various types of related biological information.

The cAMP-PKA signalling pathway is an important regulator of cell fate that controls the activity of metabolic enzymes, transcription factors and cytoskeletal proteins and is strongly associated with the onset of several endocrine and non-endocrine tumors. A fundamental characteristic of cAMP is its ability to stimulate cell proliferation in many cell types while inhibiting in others. Such ability has been related to the fact that cAMP regulates the Ras/Raf/ERK pathway, whose role in cancer onset is well known (about 25% of human cancers have a Ras mutation). Indeed the cAMP pathway is able to suppress ERK signaling through its ability to target C-Raf and conversely, to activate ERK signaling through its ability to target B-Raf [8]. The underlying inhibitory mechanism is reasonably well characterized and involves the uncoupling of Ras signaling to C-Raf. On the other hand, models to explain ERK activation by cAMP are incomplete and in addition to B-Raf the involvement of other proteins has been suggested [9,10]. Many observations regarding the cAMP ability to inhibit/stimulate proliferation by interfering with ERKs have been collected in normal or immortalized cell lines. However, recognizing the important role of both pathways in the development of cancer, is relevant to a more specific analysis of their crosstalk network also in cancer cells [11-14]. 

As previously described, the Ras pathway is able to crosstalk with the cAMP-PKA pathway by some typical signal transduction mechanisms (i.e. protein-protein interaction, protein phosphorylation). Moreover, through its ability to regulate the activity of a large number of transcription factors [15,16], the Ras pathway is able to control several transcriptional programs leading to proliferation, differentiation, metabolism, cytoskeletal reorganization and immune response. Such transcriptional programs are the result of ras-specific effectors stimulation [13,17]. Until now more than ten distinct functional classes of proteins have been involved as effectors of the small GTPase Ras, but the best studied are Raf kinases, type I phosphoinositide (PI) 3-kinases, Ral-guanine nucleotide exchange factors (Ral-GEFs), the Rac exchange factor Tiam1, and phospholipase C [18]. 

Raf and phosphatidylinositol 3-kinase (PI3K) were the first two identified Ras effectors and the main focus of research investigating Ras functions [19,20]. Raf promotes cell proliferation and differentiation through the MAP kinase (MAPK) pathway [21], at the same time as PI3K generates anti-apoptotic signalling, directly or through Akt pathway activation [22,23]. Both signalling pathways can activate two different signals distinct for their response timing. Indeed both MAPK and PI3K are able to activate phosphorylation cascades that lead, as primary effect, to post-translational modification of several substrates (membrane targets, cytosolic targets, cytoskeletal targets and nuclear targets), which rapidly activate functional processes. Early response to Ras signalling is quite fast: for instance in resting cells stimulated with mitogens, Ras-GTP level increases within 2 minutes from stimulation with serum [19]. Raf-1 undergoes transient activation within 2-3 minutes, and rapidly activates the mitogen-activated protein kinase (MAPK) cascade whose most downstream component, ERK, rapidly moves into the nucleus. Here it phosphorylates nuclear proteins, notably transcription factors [24,25] whose activity can be controlled by regulating their sub-cellular localization, expression, stability, ability to bind to other components of transcriptional complexes and to DNA, and their ability to remodel chromatin structure [26]. Transcription factors are under the control of MAPK pathway, including members of the ETS family (i.e. Ets-1, Ets-2, PU-1), MADS box family (i.e. MEF2A, MEF2C, Sp1), Zinc Finger family (i.e. GATA-2 and GATA-4), bZip family (i.e. Fra-1, c-Jun, JunB, JunD, ATF-2, c-Fos and CREB), bHLH family (i.e. c-Myc, MITF), Nuclear Hormone Receptor (i.e. PR, GR and ER) as well as other transcription factors (i.e., SMAD1, STAT1) and coregulatory proteins (i.e., CBP, p300) [15,24,25]. 

Like ERKs, Akt and other targets of PI3K signalling can phosphorylate and activate transcription factors [27]. Akt protein can control several transcription factors directly or indirectly. Direct targets are the forkhead box proteins, FOXO, and the cell cycle inhibitor, MIZ1, which are both inhibited upon AKT-mediated phosphorylation. AKT-dependent regulation of p53, nuclear factor B (NFkB), c-MYC, activator protein 1 (AP1) and beta-catenin is indirect [16]. 

Such an observation led us to re-analyze, by using a generalized workflow for data recovery and integration, available data from multiple global assays and several databases (genomics, transcriptomics, promoter analysis and literature). In particular we searched for information for genes encoding proteins of the downstream branch of the PKA pathway (starting from adenylyl cyclase and downstream) in tumor cell lines (NCI60 cells) as a function of mutational activation of different pathways (notably the Ras and PI3K pathway) in comparison with the corresponding normal tissues, with the aim to define better the connection between these pathways in cancer cells [28]. 

## Results and discussion

Gene-expression profiling has been applied extensively in cancer research. As a first step to identify regulatory mechanisms underlining gene-expression profiles it is necessary to extract, filter, cross-reference and structure information from cancer-related data sets [29]. The aim of this work has been the identification of cancer-specific specific gene expression signatures in genes encoding proteins involved in the cAMP-PKA pathway. In particular we wished to identify, if present, differences between primary normal tissues and cancer cells and search for correlation with the pathway mutationally activated in any given transformed cell line by integrating an accurate analysis of recovered data from several databases with the application of different statistical tests.

### Transformation-dependent, transcriptional remodelling of the PKA pathway encoding genes in 60 human cancer cell lines (NCI60)

The NCI60 cell collection includes cell lines derived from colorectal, renal, ovarian, breast, prostate, lung and central nervous system cancers, as well as leukaemias and melanomas (Table 1), that are most commonly used in cancer research and drug screening [30,31]. A good correlation between transcriptional profiles of the cell lines and their tumor cancer of origin [31,32] has been found for 51 out of 59 cell lines. NCI60 transcriptional profiles are available in public databases. 

**Table 1:** The cancer cell lines in the NCI60 collection sorted by tissue of origin

**Tumor Type**	**Cell lines**
Breast	HS578T, MDA-MB231, MD-MB435, MCF7, T47, MDA-N, BT549
CNS	SF295, SF359, SNB19, U251, SF268, SNB75
Colon	HCC2998, HCT116, HCT15, SW620, COLO205, HT29, KM12
Leukaemia	CCRF-CEM, RPMI-8226, HL60, MOLT4, K562, SR
Melanoma	SK-MEL2, LOXIMVI, M14, MALME-3M, SK-MEL28, SK-MEL5, UACC257, UACC62
Lung	A549, HOP62, NCI-H23, NCI-H460, EKVX, NCI-H226, NCI-H322, NCI-H522, HOP92
Ovarian	OVCAR5, OVCAR8, SKOW3, IGROV1, OVCAR3, OVCAR4
Prostate	PC3, DU145
Renal	786-0, RXF-393, A498, ACHN, CAKI-1, SNC12C, TK10, U031
Unknown	ADR-RES

Since the stabilized cell lines within the NCI60 collection represent a physiological model to study gene profiles in cancer cells, with features strongly similar to cancer tissues, we reviewed information present in public databases about the 60 cell lines and 21 normal tissues, in order to identify transformation-dependent transcriptional signatures for PKA pathway-encoding genes  (Table 2).

**Table 2:** Gene expression profiling datasets of NCI60 cell lines and normal tissues analyzed in this study

**Reference**	**Tissue of origin**	**Number of transcriptional profiles**	**GEO Number**
[31]	NCI60 cells	60	GSE5949
	Breast	0	-
[106]	CNS	2	GSE96
[107]	Colon	4	GSE6731
[108]	Blood	4	GSE1402
[106]	Lung	2	GSE96
	Skin	0	-
[106]	Ovary	3	GSE96
[106]	Prostate	3	GSE96
[106]	Kidney	3	GSE96

Gene expression profiles retrieved from the GEO Database. Dataset A (60 profiles) is made up of the NCI60 cell lines [31]. Dataset B (13 profiles) is a subset of transcriptional profiles of a diverse array of tissues, organs, and cell lines from a normal human physiological state [106]. Dataset C (4 profiles) encompasses the normal human adult samples derived from colonoscopic biopsy present in a database comprising samples of patients with Crohn's disease or ulcerative colitis [107]. Dataset D (4 profiles) contains normal control samples present in a database containing transcriptional profiles of peripheral blood mononuclear cells (PBMC) obtained by juvenile arthritis patients and healthy controls [108].

**Figure 1:**
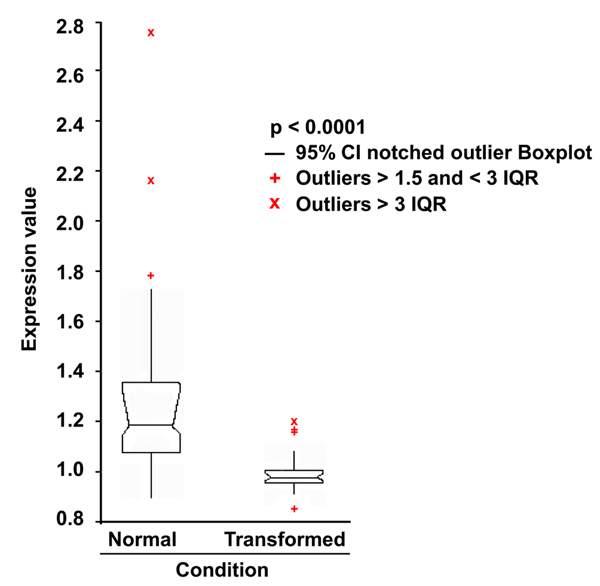
**Statistical analysis of the 41 PKA pathway-encoding genes expression in normal and transformed samples.** 81 transcriptional profiles from normal tissues and from the NCI60 cancer cell line collection, were recovered from the GEO database. After normalization (see Methods), the expression values of 41 PKA pathway-encoding genes were used to perform an ANOVA analysis (p-value 0.0001) to evaluate the statistical significance of the differences between normal and transformed samples. IQR: Interquartile Range. Outliers are also shown.

We identified and gathered the transcriptional profile for 41 genes encoding proteins involved in the PKA pathway (adenylyl cyclases -ADCY-, phosphodiesterases -PDE-, A-kinase anchor proteins -AKAP-, cAMP-dependent transcriptional factors -TF-, PKA catalytic subunits -PRKAC- and PKA regulatory subunits -PRKACR-, Table 3) and compared expression profiles of cancer cell lines with those of primary normal tissues, collected from different datasets (Table 3). To identify differences between normal and cancer samples, we performed an ANOVA analysis on the entire data set. As shown in Figure 1, distributions of expression values of genes encoding proteins of the cAMP/PKA pathway were statistically different between normal and transformed cells (p-value <0.0001), indicating that in transformed cells the PKA pathway-related genes are differentially expressed as compared to normal cells. Namely, the box plot indicates that, overall, the distribution of expression of values of transformed cells is shifted towards lower expression values. Dispersion of the distribution in transformed cells is much reduced compared to that observed in normal tissues, as if transformation events superimpose a negative regulation that largely abrogates tissue-specific regulation (i.e., the major factor responsible for dispersion of expression in normal tissues, see next paragraph). 

**Table 3:** PKA related genes identified in all the datasets shown in Table 2 and used in this study

**Probeset**	**Unigene**	**Symbol**	**Description**
33353_at	Hs.192215	ADCY1	Adenylate cyclase 1 (brain)
34686_at	Hs.481545	ADCY2	Adenylate cyclase 2 (brain)
33134_at	Hs.467898	ADCY3	Adenylate cyclase 3
39383_at	Hs.525401	ADCY6	Adenylate cyclase 6
40585_at	Hs.513578	ADCY7	Adenylate cyclase 7
36246_at	Hs.414631	ADCY8	Adenylate cyclase 8 (brain)
33800_at	Hs.391860	ADCY9	Adenylate cyclase 9
37698_at	Hs.463506	AKAP1	A kinase (PRKA) anchor protein 1
36633_at	Hs.462457	AKAP10	A kinase (PRKA) anchor protein 10
34657_at	Hs.105105	AKAP11	A kinase (PRKA) anchor protein 11
37680_at	Hs.371240	AKAP12	A kinase (PRKA) anchor protein (gravin) 12
554_at	Hs.459211	AKAP13	A kinase (PRKA) anchor protein 13
41075_at	Hs.98397	AKAP3	A kinase (PRKA) anchor protein 3
37087_at	Hs.97633	AKAP4	A kinase (PRKA) anchor protein 4
32421_at	Hs.532489	AKAP5	A kinase (PRKA) anchor protein 5
40747_at	Hs.509083	AKAP6	A kinase (PRKA) anchor protein 6
41703_r_at	Hs.486483	AKAP7	A kinase (PRKA) anchor protein 7
35138_at	Hs.199029	AKAP8	A kinase (PRKA) anchor protein 8
37886_at	Hs.399800	AKAP8L	A kinase (PRKA) anchor protein 8-like
36506_at	Hs.527348	AKAP9	A kinase (PRKA) anchor protein (yotiao) 9
36297_at	Hs.435267	ATF1	Activating transcription factor 1
37535_at	Hs.516646	CREB1	cAMP responsive element binding protein 1
32066_g_at	Hs.200250	CREM	cAMP responsive element modulator
35522_at	Hs.487129	PDE10A	Phosphodiesterase 10A
36311_at	Hs.416061	PDE1A	Phosphodiesterase 1A, calmodulin-dependent
38921_at	Hs.530871	PDE1B	Phosphodiesterase 1B, calmodulin-dependent
32418_at	Hs.487897	PDE1C	Phosphodiesterase 1C, calmodulin-dependent
666_at	Hs.89901	PDE4A	Phosphodiesterase 4A, cAMP-specific
33705_at	Hs.198072	PDE4B	Phosphodiesterase 4B, cAMP-specific
38860_at	Hs.437211	PDE4C	Phosphodiesterase 4C, cAMP-specific
38526_at	Hs.117545	PDE4D	Phosphodiesterase 4D, cAMP-specific
37676_at	Hs.9333	PDE8A	Phosphodiesterase 8A
37249_at	Hs.78106	PDE8B	Phosphodiesterase 8B
33709_at	Hs.473927	PDE9A	Phosphodiesterase 9A
438_at	Hs.194350	PRKACA	Protein kinase, cAMP-dependent, catalytic, alpha
36215_at	Hs.487325	PRKACB	Protein kinase, cAMP-dependent, catalytic, beta
36359_at	Hs.158029	PRKACG	Protein kinase, cAMP-dependent, catalytic, gamma
226_at	Hs.280342	PRKAR1A	Protein kinase, cAMP-dependent, regulatory, type I, alpha
1091_at	Hs.550753	PRKAR1B	Protein kinase, cAMP-dependent, regulatory, type I, beta
116_at	Hs.517841	PRKAR2A	Protein kinase, cAMP-dependent, regulatory, type II, alpha
37221_at	Hs.433068	PRKAR2B	Protein kinase, cAMP-dependent, regulatory, type II, beta

**Figure 2:**
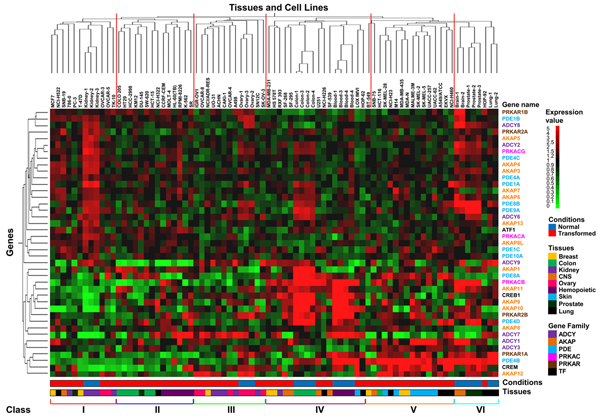
**Hierarchical clustering of the 41 PKA pathway-encoding genes analyzed in this paper.** Two-way (gene, column and cell line, row) hierarchical clustering (see Methods) of the same profiles analyzed in Figure 1. Normalized expression is colour-coded from green (poor expression) to red (strong expression). The name of each gene is colour-coded according to family to which it belongs. The 6 main classes described in the text (red lines on the top of the dendrogram and roman number bottom of the dendrogram) are shown. The distance function is based on Pearson correlation and complete linkage clustering. Legends for expression, condition, gene family and tissue of origin are shown on the right of the dendrogram.

The same data-set was then analyzed through unsupervised hierarchical clustering  (as implemented in the GeneSpring platform) that organizes genes according to the similarity or dissimilarity in expression profile, placing the cases with similar expression profiles together as neighbouring columns in the dendrogram (Figure 2).  Six different classes corresponding to the main arms of the dendrogram derived from clustering according to Tissue and cell lines (classes **I** to **III** correspond to the left main branch of dendrogram, **IV** to **VI** to the right branch) were identified. Each cell line is color-coded at the bottom according to its condition (i.e., normal, blue, or transformed, red) or the tissue of origin.  Notably, classes **II** and **V** contain only transformed cells, while only one transformed cell line clusters in class **VI**. In most cases clustering effectively separates normal and transformed cell lines of the same histological origin: for instance, normal and transformed cell lines derived from kidney cluster to class **I** and **III**, hemopoietic normal and transformed cell lines to **IV** and **II**, colon cancer cells are in class **II** while normal colon in class **IV**, respectively (Table 4).  Class **I** and **IV** contained cancer lines of several histological origin, while class **II** was enriched for cancer cells from colon and blood, class **III** for ovary and kidney and class **V** for lung, respectively (Table 4). These results indicate that regulation of the PKA pathway is tissue-dependent, in keeping with the pleiotropic and tissue-specific phenotypes regulated by intracellular cAMP. They also suggest that transformation transcriptionally remodulates the PKA pathway, so that in most cases expression profiling of genes encoding proteins of the cAMP-PKA pathway is quite different in cancer cells as compared to their normal counterparts. Interestingly in class **IV**, which comprises all the colon and hemopoietic normal samples, we observe strong expression of few genes (AKAP9-11; PDE4D; PRKCB and PRKAR2B; CREB1- colon sample- and AKAP9-11; PDE4B and PDE8A; PRKCB and PRKAR1A; CREB1 and CREM- hemopoietic sample-) as compared to their transformed counterparts, in which the same genes appeared poorly expressed (class **II**). In human colon carcinoma cells it has been reported that PRKAR2B overexpression suppresses neoplastic cell growth [33], consistently with the notion that abnormal expression of isoforms of PKA regulatory subunits may be involved in neoplastic transformation. Moreover in several models of hemopoietic malignancies, it has been shown that induction of cAMP/PKA pathway stimulates leukemia cell differentiation (event associated to the relapse of the disease) or lymphoma cells apoptosis [34,35].

**Table 4:** Correlation between PKA related gene patterns and tissues

**Class**	**Normal**	**Transformed**
I	Kidney 3/3	Breast 2/8 Lung 1/9 CNS 1/6 Kidney 2/8 Prostate 1/2 Ovary 2/6

II		Colon 6/7 Prostate 1/2 Lung 1/9 Hemopoietic 6/7

III	Ovary 3/3	Ovary 4/6 Breast 1/8 Kidney 5/8

IV	Colon 4/4 Hemopoietic 4/4	Breast 2/8 Kidney 1/8 CNS 4/6 Lung 2/9 Skin 1/8

V		Breast 3/8 CNS 1/6 Colon 1/7 Skin 7/8 Lung 4/9

VI	CNS 2/2 Prostate 3/3 Lung 2/2	Lung 1/9

Six different classes, corresponding to the main arms of the dendrogram derived from clustering according to "Tissue and cell line", were identified. The number on the right of each tissue represents the number of samples belonging to a class as compared to the total sample analyzed.

### Analysis of mutational status of the NCI60 cell lines and correlation with tissue-specific PKA pathway gene regulation

In the previous paragraph we have shown that a different and a tissue-specific pattern of expression of the PKA pathway encoding genes between normal and transformed samples does exist. Moreover, we observed that a similar pattern is common to different tissues, both in normal and transformed samples. While in normal tissues such a finding may be justified by a common histological origin or by the PKA pathway regulating a common intracellular process (i.e. differentiation, metabolism), in transformed samples, in which the correct regulation of the PKA pathway is lost, such similar gene regulation can suggest a transformation or a mutation-dependent gene regulation. 

For this aim, we determined the mutation status of the NCI-60 panel of human cancer cell lines, identified the pathway in which such mutations were involved and correlated the mutation status and pathway altered in the transformed cells with transcriptional profiling data. The 60 cell lines were sorted according to mutational status, using the information provided by Catalogue Of Somatic Mutations In Cancer (), and divided into four groups based on the carried mutation as follows (Table 5):

1) Cell lines carrying mutations able to interfere with the Ras pathway (i.e., mutations in genes encoding Ras, B-Raf, ERBB2, PDGFRA, referred to as Ras), 29 cell lines;

2) Cell lines carrying mutations able to interfere with PI3K-Akt pathway (i.e., mutations in genes encoding PI3KCA, PTEN and Lkb1, referred to as PI3K), 13 cell lines.

3) Cell lines carrying no somatic mutations interfering with the two above pathways (i.e., mutations in genes encoding CDKN2A, p53, referred to as Other Mutation), 14 cell lines;

4) Cell lines for which the presence of somatic mutations interfering with the above pathways has not been searched, referred to as Not Tested), 4 cell lines.

**Table 5:** NCI60 cell lines with predicted active pathways by mutational analysis

**Tumor Type**	**Ras**	**PI3K**	**Other Mutation**	**Not Tested**
**Breast**	HS578T, MDA-MB231, MD-MB435	MCF7, T47,	-	MDA-N, BT549
**CNS**	-	SF295, SF359, SNB19, U251	SF268, SNB75	-
**Colon**	HCC2998, HCT116, HCT15, SW620, COLO205, HT29	KM12	-	-
**Leukaemia**	CCRF-CEM, RPMI-8226, HL60, MOLT4, K562	-	-	SR
**Melanoma**	SK-MEL2, LOXIMVI, M14, MALME-3M, SK-MEL28, SK-MEL5, UACC257, UACC62	-	-	-
**Lung**	A549, HOP62, NCI-H23, NCI-H460	-	EKVX, NCI-H226, NCI-H322, NCI-H522	HOP92
**Ovarian**	OVCAR5, OVCAR8	SKOW3, IGROV1	OVCAR3, OVCAR4	-
**Prostate**	-	PC3, DU145	-	-
**Renal**	-	786-0, RXF-393	A498, ACHN, CAKI-1, SNC12C, TK10, U031	-
**Unknown**	ADR-RES	-	-	-

The 60 cell lines reorganized in 4 categories, as described in the text, on the basis of the most representative mutation of each cell line:

Cell lines carrying mutations able to interfere with Ras-Raf-MAPK pathway (i.e., mutations in genes encoding Ras, B-Raf, ERBB2, PDGFRA, referred to as Ras);

Cell lines carrying mutations able to interfere with PI3K-Akt pathway (i.e., mutations in genes encoding PI3KCA, PTEN and Lkb1, referred to as PI3K);

Cell lines carrying no somatic mutations interfering with the two above pathways (i.e., mutations in genes encoding for CDKN2A, p53, referred to as Other Mutation);

Cell lines for which the presence of somatic mutations interfering with the two above pathways has not been searched, referred to as Not Tested.

**Figure 3:**
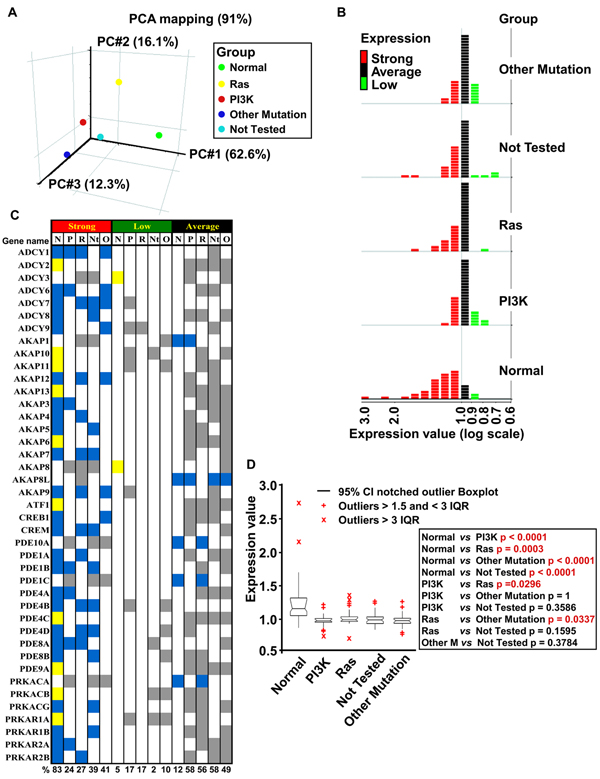
**Identification of differentially regulated genes in normal and transformed samples. (A)** Samples were sorted in five groups according to mutational activation: green, normal; yellow, Ras; red, PI3K; blue, Other Mutation; cyan, Not Tested. Principal Component Analysis (PCA) performed on 41 PKA pathway-encoding genes for normal samples and the four classes of mutation-dependent samples. Each sphere represents the comparative averaging of the 41 genes for each pathway identified by mutational analysis. **(B)** For each of the 5 groups described in **(A)**, the 41 PKA-encoding genes were clustered, relative to their level of expression, in three subgroups: Strong (>1, red), Average (=1, black) and Low (<1, green). **(C)** Gene list according to expression level and mutational group of the three subgroups previously indicated, divided for each sample. Color-coding is as follows: blue, common between normal and at least one transformed sample; yellow, specific for normal samples; grey, specific for transformed samples. Percentage of regulated genes for each subgroup is shown at the bottom. **(D)** ANOVA analysis to evaluate the statistical significance of the differences between the five classes of samples described in **(A)**. The right inset shows p-value of the pair-wise comparisons. Statistically significant differences are indicated in red. IQR: Interquartile Range. Outliers are also shown.

To assess overall data quality and visualize relations and differences between the aforementioned transformed and normal samples, we applied dimensional reduction through principal component analysis (PCA). A three-dimensional PCA plot of all expression data (accounting for 91% of variance) is shown in Figure 3A. PC1  (x axis) effectively separates the normal group from the four groups of transformed cells. PC2  (y axis) effectively separates the Ras group from the others, while PC3  (z axis) best separates the Other Mutation group from the others.  Overall, the Ras group appeared to segregate the most from the other groups.

In Figure 3B, the 41 genes encoding proteins involved in the PKA pathway were sorted according to their relative level of expression and color-coded in the graph according to expression: strong (red, value >1), average (black, value=1) and low (green, level <1). These three series were crossed with the groups described above, namely Normal, Ras, PI3K, Other Mutation and Not Tested. In Normal tissues, expression of 83% of the genes was classified as Strong, a value 2-3 fold higher than those observed in the different transformed groups (27-41%). Overall, in the transformed groups, expression of most PKA pathway-encoding genes was classified as Average or Low, with the exception of the Ras group, in which only one gene was scored as low.

Expression of PKA pathway-encoding genes was further classified as follows (Figure 3C): genes with similar level of expression between normal and at least one transformed group (blue color), genes whose expression level is different between the normal and transformed groups (yellow color) and genes with similar expression level among the different transformed groups (grey color). Such a classification allowed us to pinpoint genes, such as ADCY2 and AKAP13 whose expression is strong only in the Normal group. More interestingly, expression of a few genes, such as ADCY3 and AKAP8 was strong only in members of the transformed groups, despite overall reduction in expression of the PKA pathway-encoding genes observed in transformed samples. 

These results were further confirmed by pair-wise ANOVA analyses (Figure 3D), in which the distribution of expression values of genes encoding proteins of the cAMP/PKA pathway were found to be statistically different between normal and each group of transformed cells (p-value between 0.0001 and 0.0003). Notably, the difference in distribution between the Ras group and the PI3K and Other Mutation groups was also statistically significant, unlike the difference with the Not Tested group. This suggests that cells in this group may be biased for mutations within genes encoding proteins of the Ras pathway. 

**Figure 4:**
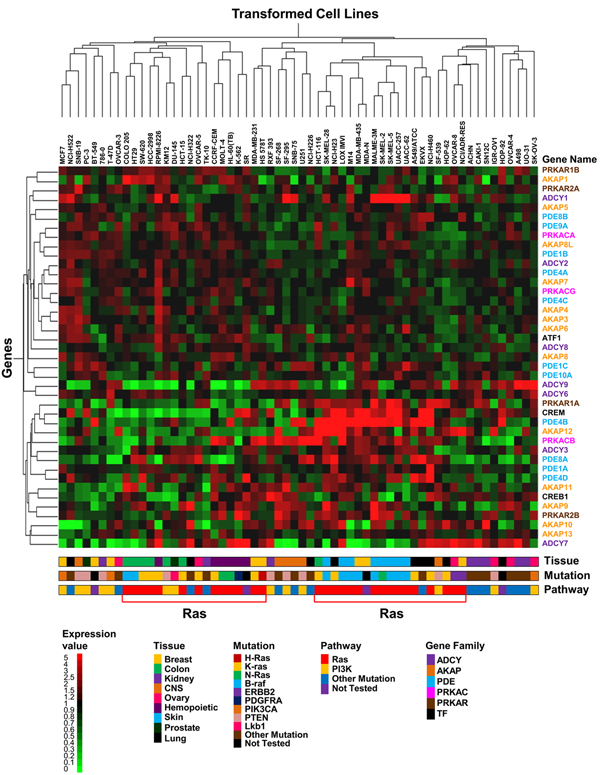
**Hierarchical clustering of the 41 PKA pathway-encoding genes in transformed samples.** Two-way (gene, column and cell line, row) hierarchical clustering (see Methods) of the profiles from the NCI60 collection only. Normalized expression is colour-coded from green (poor expression) to red (strong expression). The distance function is based on Pearson correlation and complete linkage clustering. The name of each gene is colour-coded according to the family to which it belongs. Legends for expression, condition, gene family and tissue of origin are shown on the right of the dendrogram. The data have been organized on the basis of the tissue of origin of the cancer (Tissue), the specific oncogenic mutations identified in each cell line (Mutation), the putative altered pathway by the specific mutations (Pathway) and the gene family.

To reveal gene expression changes relate to mutation status of the 60 cell lines, and better interpret the results of PCA and ANOVA, a hierarchical clustering was performed. The resulting dendrogram is shown in Figure 4, in which each cell line is color-coded at the bottom according to its tissue of origin -row labeled tissue-, mutated gene -row labeled mutation-, inferred pathway activated by mutation -row labeled pathway-. A robust association between the transcriptional profiles and mutations in the Ras pathway was observed (indicated as Ras, red color). Two cell lines of the Not Tested group were interdispersed within the Ras group, indicating that these two lines are most likely responsible for the lack of statistical difference between the Ras and the Not Tested group (see above). Comparison of the Tissue and Pathway categories indicated that within the two Ras sub-clusters, some tissue-specificity is conserved. Indeed, the left cluster, comprising a total of 18 cell lines, was characterized by 6 colon cancers and 6 leukemias of which 5 on 6 were mutated in Ras pathway. Similarly the right cluster, comprising a total of 19 cell lines, was characterized by 8 melanomas and 5 lung cancers of which 7 on 8 were mutated in Ras pathway for melanoma and 4 on 5 for lung cancer. The other sub-clusters, comprising all the remaining cell lines and the other three groups of mutations and consequently of pathways, were more dispersed along the clustergram. Together, these results indicate that transformation events modulate transcriptional regulation of genes encoding proteins of the PKA pathway and that mutational activation of the Ras pathway originates a distinguishable signature, in comparison with mutational activation of the other genes studied in this report. Such a distinguishable signature is particularly noticeable in melanoma cells, in which strong expression of a gene set encoding a complete functional PKA pathway module (ADCY3; PDE4B, PDE4D and PDE8A; AKAP12; PRKAR1A and PRKAR2B; PRKACB; CREM) is observed, suggesting a deregulated cAMP signaling. Moreover, analysis of expression values for PRKAR1A and PRKAR2B genes indicated the presence in melanoma cells of a high R1/R2 ratio, that has been associated to melanocyte proliferation [36]. 

### Promoter analysis: finding correlation between oncogenic pathway, transcriptional profiles and promoter regulation

**Figure 5:**
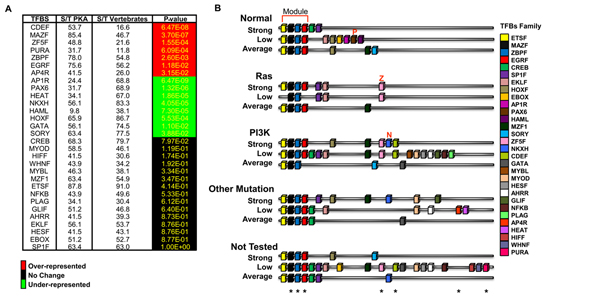
**TFBS identification by using the enrichment as parameter. (A)** The panel shows for each TFBS, recognized as relevant (present in ≥ 70% of the promoters of 41 PKA pathway-encoding genes) the percentage of promoters in our collection that contain the motif as compared to Matrix Family Library on vertebrates. This percentage has been calculated by dividing the total number of promoters containing the motif (S) by the total number of promoters (T). Color-coding scheme on the right of the panel. **(B)** Schematic representation of the TFBSs (color-coded as shown on the right of the panel) identified in the promoters of the 15 subgroups described in the text and in Figure 3. Each cartoon represents the promoter structure resulting from the average of the TFBS identified in ≥ 70% of the gene promoters for each subgroup. The asterisks on the bottom of the cartoon indicate the over-represented TFBS, as scored in panel **A**, for all the 41 PKA pathway-encoding genes.

Genes involved in the same pathway or transcriptionally co-regulated are likely to share similar promoter features. To test this hypothesis in our model, the 15 groups previously established (see Figure 3), containing coregulated genes for each group, were used for promoter identification and analysis. Using a series of biocomputing procedures and statistical processes (see Methods and the Figure), we identified Transcription Factor Binding Sites (TFBSs) conserved within the promoters (operationally defined as regions spanning 500 nt upstream and 100 nt downstream from the transcription start site) of the 41 PKA pathway-encoding genes. Genes were sorted in the 15 groups indicated in Figure 3B and 3C, and each group separately analyzed. In this first analysis (Fig. 5A), each TFBS was scored as either absent or present, regardless of the number of copies present within a given promoter. This analysis permitted the identification of 30 TFBSs enriched in the promoters of the 41 PKA pathway-encoding genes whose frequency of occurrence i.e., the ratio between the promoters that contained the specified motif  (S) and the 41 promoters in our collection (T) was compared with the frequency of occurrence within vertebrate genomes (computed using the promoter Library Matrix Family of vertebrates that comprises 260.000 vertebrate promoters). Statistical analysis indicated that of these 30 TFBSs, 7 were over-represented (red color) and 9 under-represented (green color). The remaining showed the same frequency of occurrence found in the whole vertebrate genome collection. 

A *consensus* representation for the promoter structure of each subgroup of coregulated genes was drawn by taking into account the 30 TFBSs present in at least 70% of the genes within each subgroup (Figure 5B). Surprisingly, the vast majority of these consensus promoters (13 out of 15) showed a common module (upper part, module), comprising 4 TFBSs: ETSF, MAZF, ZBPF and EGRF, 3 of which are over-represented in our collection (over-represented motifs are indicated by an asterisk at the bottom of the figure). This strongly suggests a functional implication of these TFBSs in expression of PKA pathway-encoding genes. Other interesting features indicated by this analysis include the identification of binding sites for PAX6 (indicated by red P) and ZF5F and NKXH (indicated by red Z and N respectively) only in *consensus* promoters of some genes within the normal or transformed group, respectively.

**Figure 6:**
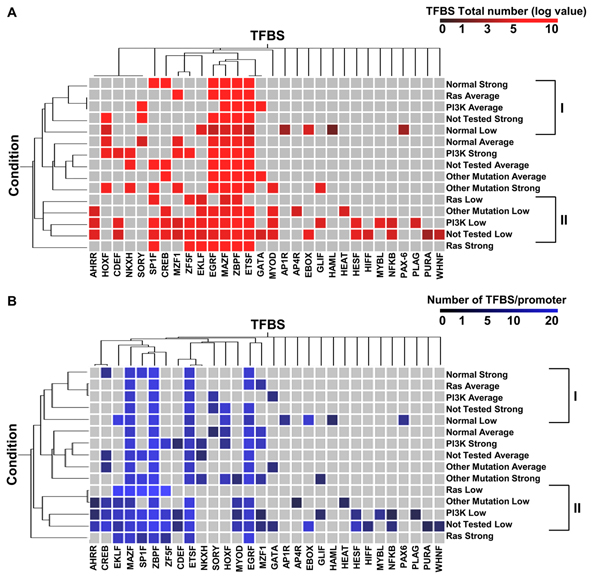
**Hierarchical clustering of TFBSs present in the promoters of the 41 PKA pathway-encoding genes, according to total number and frequency.** Two-way (TBFS, column and expression subgroup, see Figure 3, row) hierarchical clustering of the TFBS present within the promoters of the 41 PKA pathway-encoding genes. Clustering was run according to the total number of TBFS present in each group (panel **A**) or to the frequency, i.e. the total number of a given TBFS divided by the number of promoters (panel (**B**). The color-coding scale is shown at the top of each panel. The distance function is based on Pearson correlation and complete linkage clustering. The two classes, corresponding to the main arms of the dendrogram, derived from clustering according to "Condition" are shown on the right of each dendrogram.

Another feature that may be critical in the identification of enriched elements is the number of copies of a given TFBS within a promoter. In fact, it has been documented that the presence of multiple copies of *cis*-elements in promoters, particularly when clustered, makes transcriptional activation stronger [31,32]. For this reason, total number and frequency (number of each TFBS/promoter) of the 30 TFBSs previously identified, was scored within each of the 15 subgroups and classified by hierarchical clustering (Figure 6A and 6B, respectively). Analysis using both criteria confirmed the results reported in Figure 5: the presence in promoters of all subgroups of a TFBS module comprising ETSF, MAZF, ZBPF and EGRF. Clustering according to Regulation in Figure 6A show that all promoters of genes characterized by low expression transformed samples cluster together (class II). Promoters belonging to genes with strong expression in the Ras group cluster in a completely independent arm (lower part of the dendrogram), opposite to where cluster promoters belonging to genes with strong expression in the Normal group (class I). Additionally, clustering by frequency highlighted the specific enrichment of EKLF in genes with low expression. Clustering according to both criteria indicated that Normal samples clustered in a different way as compared to transformed samples (upper part of the dendrogram) and that the PI3K, Other Mutation and Not Tested samples were more interspersed along the dendrogram and confirmed that the Ras category showed a different promoter composition as compared to other categories, in keeping with the PCA analysis presented in Figure 3.

### Data mining for PKA pathway-related gene promoters

As previously described, computational analysis of our promoter collection, permitted the identification of some TFBS that are able to characterize in a specific manner normal and transformed samples. To confirm some of our computational results, we interrogated several databases and searched in the literature for studies on promoter structure of PKA pathway-encoding genes. Experimental studies, using one or more molecular approaches including EMSA, Chromatin Immunoprecipitation and transactivation assay, have been found for 16 PKA pathway-encoding genes: PRKAR1A, PRKAR1B, PRKAR2B, PRKACA, AKAP1, AKAP8, AKAP9, AKAP10, AKAP12, ADCY8, ADCY9, PDE4B, PDE4C, PDE4D, CREB and CREM. This subset of genes was re-analyzed as described above and the obtained results were compared with literature data (Table 6). In total, 36 TFBSs have been experimentally identified: 20 of these (i.e. 55%) have been predicted by our computational approach and for two genes alone (AKAP9 and PRKAKA), none of the experimentally identified sites was identified by the computational approach that overall identified a much higher number of sites compared to those retrieved from literature. The biological significance of the presence of the identified TFBS and of their relationship with oncogenic mutations, notably in the Ras pathway, is proposed below. 

**Table 6:** Comparison between computational data and literature data

**GeneName**	**Computational data**	**Literature data**
**ADCY8**	HOXF, ETSF, SORY	CREB
**ADCY9**	ZF5F, SP1F, ZBPF, MAZF, EKLF, EGRF, **EBOX**, CDEF, WHNF, AHRR, ETSF, HESF	**c-Myc/EBOX **
**AKAP1**	MYOD, EKLF, MAZF, EGRF, ETSF, HOXF, AP1R, **EBOX**	**c-Myc/EBOX **
**AKAP8**	SP1F, EKLF, ZBPF, EBOX, ZF5F, HIFF, MAZF, EGRF, ETSF, AHRR, **CREB**	**CREB**
**AKAP9**	ZBPF, EGRF, EKLF, ETSF, HOXF, SP1F, MAZF, GLIF, HOMF, NKXH, CREB	c-Myc/EBOX
**AKAP10**	ETSF, SP1F, HESF, **EBOX**, ZBPF, MAZF, MYBL, MYOD, AP4R, EGRF, CREB, MZF1	**c-Myc/EBOX **
**AKAP12**	**EBOX**, EGRF, SORY	**c-Myc/EBOX**, SRF/SRFF
**CREB1**	SORY, **SP1F**, **NFKB**, ETSF, ZBPF, MYOD, EKLF, AP4R, MZF1, HICF	Myc/EBOX, **Sp1/SP1F**, **NFkB/NFKB**
**CREM**	ZBPF, EGRF, SP1F, MAZF, EKLF, ETSF, **CREB**, WHNF, HESF, EBOX, MYBL, HIFF, AHRR	**CREB**
**PDE4B**	ETSF, SORY, HOXF, ZBPF, HEAT, **CREB**	**CREB**
**PDE4C**	ZBPF, ETSF, **CREB**, GLIF, MAZF	Myc/EBOX, **CREB**
**PDE4D**	SORY, HOXF, NKXH, ETSF, **CREB**, GATA, MYOD	**CREB**
**PRKACA**	EKLF, ZBPF, EGRF, MZF1, NKXH, ETSF, MAZF, SORY, HOXF	USF1/EBOX, USF2/EBOX
**PRKAR1A**	ETSF, **CREB**, ZBPF, EBOX, EGRF, SORY, GATA, MYBL, MYOD, HIFF, **SP1F**, HESF	AP1/AP1F, AP2/AP2F, **Sp1/SP1F, CREB,** FOXC2, FOXD, FOXD2
**PRKAR1B**	EKLF, MAZF, HESF, PLAG, ZBPF, EBOX, **EGRF**, MYOD, MZF1, AHRR, ETSF, HIFF, SP1F, **AP1F**	**Jun/AP1F**, p53/P53F, Oct-1/OCT1, **Egr1/EGRF** Pax1/PAX1
**PRKAR2B**	ZBPF, ZF5F, EGRF, EKLF, MAZF, HESF, **SP1F**, CREB, **EBOX**, ETSF, **NF1F**	**Sp1/SP1F, NF-1/NF1F, Myc/EBOX,** CEBPbeta/CEBP** USF1, USF2/EBOX**

The table shows the transcriptional factor families identified by computational analysis and the transcriptional factors/transcriptional family identified by analysis of literature data. The factors identified by both analysis are shown in **bold**.

PKA type I regulatory subunit A (PRKAR1A) expression has been studied in different cellular models by analyzing its mRNA expression and by using its putative promoter region. In its promoter, binding sites for activator protein-1 and 2 (AP-1 and AP-2) and Sp1 [37] have been identified. Moreover, a more recent work showed a direct activity of FOX family (FOXC2, D1 and D2) transcriptional factors members in the regulation of PRKAR1A expression both at transcriptional and at post-transcriptional levels [38,39].

The promoter of PRKAR1B has been identified and studied in human and mouse: binding sites for Jun and p53 (human) and Oct-1, Egr1 and Pax1 (mouse) have been found. These binding sites have been experimentally verified by Electrophoretic Mobility Shift Assay, functional analysis and Northern blot [40,41].

PRKAR2B promoter has been studied in particular in Sertoli cells (human). Some reports identified binding sites for Sp1, NF-1, Myc, C/EBPbeta, able to induce the PRKAR2B promoter, USF1 and USF2. Interestingly, overexpression of USF2, but not USF1, led to inhibition of both cAMP- and C/EBPbeta-mediated induction of PRKAR2B [42-44].

The promoter of Protein kinase, cAMP-dependent, catalytic, alpha (PRKACA) has been identified both in humans and mouse, but little information has been produced for human promoter. Indeed, one paper describes the presence of binding sites for USF1 and USF2 transcription factors [45].

AKAP1, AKAP9 and AKAP10 promoters contain binding sites for c-Myc as shown by computational analysis and ChIP experiments in several human cell lines [46,47]. Moreover, a single study indicates the presence in the promoter of AKAP12 of binding sites for Serum Response Factor transcriptional factors [48] and more recently for Myc.

ADCY9 promoter contains binding sites for c-Myc as shown by an experimental approach [49].

Several promoters of genes encoding phosphodiesterase proteins have been isolated and to some extent studied. All the studies have been performed on sequences of human promoters and in particular the PDE4B, PDE4C (both present in our collection of PKA pathway related genes), and PDE5A, PDE6A, PDE6B and PDE7A promoters (not present in our gene list) have been better characterized. In the PDE4B promoter, binding sites for CREB have been found [50]. In PDE4C promoter, binding sites for Myc have been found [47]. In the PDE5A promoter, binding sites for Jun and AP-2 have been found [51,52]; in PDE6A and PDE6B promoters, binding sites for Sp1 [53] and Sp4 [54,55] respectively and in PDE7A promoter, Ets2 and NFkB1 binding sites [56].

The cyclic AMP response element (CRE)-binding protein CREB promoter has been identified in human, mouse and rat. Analysis done on human promoter, experimentally confirmed, identified binding site for c-Myc [57] and Sp1 [58]. Further information about such promoter has been produced in mouse and rat cells which allowed the identification of binding site for NfkB [57].

An important regulative mechanism of the PKA pathway is feedback control. Indeed as well as the cAMP produced by Adenylyl Cyclases, activate PKA kinase activity, PKA is able to inhibit the pathway, activating by phosphorylation the Phosphodiesterases, which ultimately induce hydrolysis of cAMP switching off the pathway. Moreover a huge amount of data has been published regarding the ability of PKA to activate specific transcription factors by phosphorylation: cyclic AMP response element (CRE)-binding protein CREB, the cAMP response element modulator (CREM), the activating transcription factor 1 (ATF-1) and a repressor, ICER (inducible cAMP early repressor) [59] that, to a certain extent, has been shown to regulate PKA pathway-related genes transcription. Some of the promoters, already discussed above, have been shown to have CRE binding sites. Moreover, two interesting recent publications, have identified and characterized in different cellular contexts and by several approaches, through a genome-wide approach, target genes that are regulated by CREB [60,61]. The authors have identified and proved by ChiP analysis (PRKAR1A, PDE7B) the presence of CRE site in PRKAR1A, in PDE7B, AKAP8, PDE4C and ADCY8. In the latter case they did not observe binding by Chip analysis, but another report has shown that its activation is mediated specifically via the canonical CRE site [62]. Binding sites for CREB1 have been found in PDE7A [56], PDE4D [63], CREM [64] and experimentally confirmed. Moreover analysis of the promoter of CREB gene showed the presence of several CRE binding sites [65,66]. 

Most of AP-1 (i.e. Jun), AP-2 and Sp1 transcription factors are involved in growth-related signal transduction pathways, among which Ras is a main actor, and their over-expression can have positive or negative effects on proliferation [67-71]. Indeed Sp family has been shown to be regulated by post-translational mechanisms by Ras pathway [72,73] as well as Ets1 and Ets2 [74,75] and NFkB [76-78].

Egr-1 is an early responsive gene linked to mitogenic stimulation directly regulated by MAPK pathway [79-82]. Moreover for Myc [83,84], C/EBPbeta [85] and NF-1 [86] a large amount of data about their correlation with Ras pathway has been reported. Each of these transcriptional factors has been associated with several cellular responses (proliferation, survival, apoptosis) and transformation as is the case of the PKA pathway as well. Therefore it is possible that mitogenic signal through Ras and the regulation of such transcription factors, modulates the expression of PKA pathway related genes. 

An important role, in the activation of the CREB family transcription factors, is played by stimuli which are able to induce their phosphorylation and consequently their activation. In fact as reviewed in [65] not only the protein kinase A is involved in this function but also several growth factors (NGF, FGF, IGF-I, PDGF, EGF), survival signals and hypoxia that often activate the Ras pathway, pointing to an essential role of the latter pathway also in gene transcriptional regulation of PKA pathway-encoding genes by transcription factors of the CREB family. 

## Conclusions

By using a generalized workflow for data recovery and integration that combines accurate analysis of recovered data from several databases with the application of different statistical tests we have been able to correlate strong transcriptional repression of genes encoding proteins of the cAMP/PKA pathway in transformed samples of different genetic origin (i.e., bearing mutations in different pathways). This finding prompted us to compute *consensus* promoters, whose composition was specifically enriched for different transcription factor binding sites (TBFS). Comparison of TFBS computationally identified in the *consensus* promoters with TBFS experimentally identified by a variety of techniques, shows a good agreement. Indeed, by lowering the stringency used in the workflow, some of the TFBS missed by higher stringency analysis (false negatives) were recovered, in keeping with the notion that intersection of different data sets and/or techniques decreases both noise and the number of hits.

**Figure 7:**
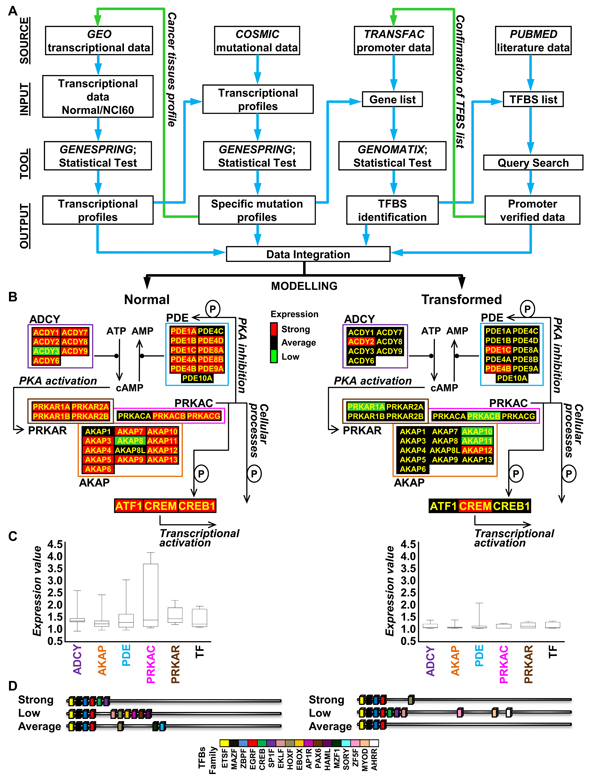
**Flowchart of our web-based and statistical strategy used to elucidate the relation between PKA encoding genes transcriptional profiles and oncogenic mutations. (A)** Flow chart of our web-based and statistical strategy with indication of some of the databases (Source) used, the type of data analyzed (Input), the specific program and statistical test (Tool) used and the result obtained (Output). **(B)** Graphical representation of the block diagram summarizing functional interconnections within the PKA pathway module with indication of the expression level (geometric mean) of each gene belonging to the network -Strong (red), Average (black) and Low (green)- as identified by our analysis both in normal (B, left) and transformed samples (B, right). **(C)** Boxplots of the expression of PKA pathway-encoding genes in normal (C, left) and transformed (C, right) samples, grouped for functional classes (ADCY: adenylyl cyclase; AKAP: A-kinase anchor protein; PDE: phosphodiesterase; PRKACR: PKA regulatory subunit; PRKAC: PKA catalytic subunit). The represented value is the median. **(D)** Schematic representation of the TFBSs (color-coded) identified in the promoters of PKA pathway-encoding genes of normal and transformed samples. Each cartoon represents the promoter structure resulting from the merge of the TFBS identified in ≥ 70% of the gene promoters of all normal samples and transformed samples.

The workflow we have followed is summarized in Figure 7 and detailed in Methods section. As the number of sites hosting curated transcriptional profiles increases, more and more data to be used as starting point become available. We used the GEO database to recover data from the NCI60 cell collection (cancer samples) and matching normal tissues and to which specific statistical tests (i.e. ANOVA, Hierarchical clustering) were applied. By using the COSMIC database, which gives information about the mutational status of the NCI60 collection, we could sort the NCI60 cell lines in 4 subgroups with mutational activation of genes encoding components of the Ras pathway, of the PI3K pathway, of other pathways or for which no information was available. Such a sorting allowed us to uncover an hitherto unrecognized oncogene-dependent pattern of regulation of 41 genes encoding components of the cAMP/PKA pathway (Figure 7B and 7C).  The transcriptional profiles for transformed cells within one of the identified subgroups may then be used as a new query to GEO database (green arrow), in order to correlate and confirm, i.e. in cancer tissues, the oncogene-dependent pattern identified.

Deregulation of transcriptional programs, such as that identified for PKA pathway-encoding genes, may be considered a direct consequence of a deregulated activity of transcription factors. The TRANSFAC database was used with a high stringency threshold, to identify the regulatory sequence in co-regulated genes with high confidence, improving the deduced linkages between transcription factors and the regulated genes.  Using this approach, we demonstrated that in all PKA encoding genes TFBSs for ETS, MAZ, ZBP and EGR transcription factors are present (Figure 7D) and that specific subsets of TFBS are present in the normal and transformed samples. The number of TFBS identified by computational analysis was higher than those that could be retrieved from literature as experimentally determined. This observation was to some extent expected because of limited literature reference availability, complexity to retrieve data, difficulty to analyze data from several origins, and the lack of powerful data analysis and integration tools. Under these less-than-ideal conditions, a dedicated tool such as the TFBS database, can be extremely powerful, allowing predictions that are amenable to experimental verification, should this be necessary. As discussed above, most of the false-negatives that failed to be detected by our computational approaches could be recovered by appropriately lowering the stringency of analysis.  

In Figure 7B transcriptional expression of PKA pathway encoding genes is color-mapped (geometric mean, Strong expression, red, Average expression, black and Low expression green) on a block diagram summarizing functional interconnections within the PKA pathway module. A general and balanced co-regulation of both positive and negative regulators of the cAMP/PKA pathway is apparent in both normal and transformed samples. Notably, in normal cells variability in expression is maximal for genes encoding the catalytic subunit of PKA.  Because of the pleiotropic role of the PKA pathway (including stimulation of growth and differentiation in many cell types, such as somatotrophs, thyrocytes, melanocytes, ovarian follicular granulosa cells, keratinocytes, nervous, muscle and blood cells and adipocyte and the important role of such pathway in the regulation of the function of tissues as kidney, ovary, brain, and prostate), strong expression in normal tissues is expected [8,87,88]. It should also be remembered that cross-talk between the PKA pathway and oncogene-mediated pathways can also take place at post-transcriptional levels. For example, several Authors reported the ability of oncogenic and viral Ras proteins to either stimulate [89-91] or inhibit [92-94] ADCY activity in different cell lines (thyroid, epithelial, kidney, fibroblast). Moreover an involvement of MAPK or PI3K pathways in the regulation of PDE activity has been reported, suggesting that mitogenic stimulation may positively regulate PDE4 expression directly [95], confirming our transcriptional results, or by post-translational mechanisms in which p42(MAPK) phosphorylation activity has a relevant role in their regulation [96]. Another important post-transcriptional mechanism that links Ras or PI3K pathways to cAMP/PKA pathway is the positive and negative control of CREB activity by a phosphorylation [97,98]. Moreover, it has been reported that cAMP is able to induce proliferation rather than growth inhibition, in several tumors where oncogenic activation of B-Raf has been identified (i.e., melanoma and thyroid cancer) [8]. Nevertheless, the general and coordinated down-regulation of essentially all genes of the pathway in transformed cells (as compared with normal tissues) suggests that at least one PKA-mediated function needs to be reduced substantially in order to express the transformed phenotype. Although at this stage it is too early to propose specific hypotheses, it is intriguing to remember that PKA has been ascribed a role in activating mitochondrial respiration and decreasing ROS production [99,100], thus effectively counteracting mitochondria dysfunction that is found associated with increased glycolysis (Warburg effect, [101-103]) in many cancer cells. On the other hand, a reduction in oxidative phosphorylation that will decrease ATP supply, as substrate of adenylate cyclase, may result in a decreased cAMP production without relevant changes in the level of the enzymes (and possibly therefore of their gene expression). 

It is expected that deeper computational integration of transcriptional data with other genome-wide findings, including -but not limited to- proteomics, interactomics and metabolomics, will allow a better extraction of hidden information. We propose that such data integration can be further applied to examine the topology of biological networks, to provide information on directionality of interactions, and create wiring diagrams that better depict the functional outcome of component-component relationships. Together, these strategies should facilitate a systems approach to modular biology.

Thus systems biology can be approached by perturbing the suspected components of a given cellular process, monitoring the responses, integrating the data and modeling the biological process in question [104]. By applying a single "omic" approach, able to sample a "horizontal" slice (i.e. across all genes or gene products) of a multidimensional space, the knowledge of a system can be expanded from a single gene to a network of genes, which can be regarded as a basic model for the system. When genes or proteins in this network are systematically disrupted, responses from other parts of the network can be recorded and the data obtained can be incorporated into the basic model. However sampling a single dimension of a complex space will undoubtedly provide relevant information, but may not highlight the major regulatory features. Therefore, a wiring diagram that depicts the direction of interactions in the network and the behavior of each of these components can be constructed to better represent the relationships between the components [104]. The example shown in Figure 7 illustrates how our current knowledge of a biological system can be expanded and a model built based on integrated "omic" information. Ultimately, development of such computational methods and their recursive integration with genome-wide and hypothesis-driven experimental investigations that also take into account post-translational and substrate-dependent mechanisms controlling the cAMP/PKA pathway activity, should reconcile experiments from different experimental systems (cell cultures, animal models and human tumor samples) and contribute to explain at an integrative, systems level how the cAMP-PKA pathway is affected by oncogenic processes originated by mutational activation of signal transduction.

## Methods

### Data recovery and normalization

Gene expression data of NCI60 cell lines and normal tissues samples were downloaded from the Gene Expression Omnibus (GEO) at the National Center for Biotechnology Information (NCBI) website () [105]. In particular, gene expression profiles of NCI60 cell collection (cancer samples) were recovered from GEO database (**GSE5949, **[31]) in which the experimental data were obtained by using the Affymetrix HG-U95Av2 oligonucleotide array platform. For the analysis only results obtained by oligonucleotide arrays were considered, because this platform uses a different method to evaluate mRNA expression as compared to cDNA array platform. Therefore, also for normal tissue samples, the data used for the comparative analysis, were recovered from transcriptional profiles produced by using U95Av2 oligonucleotide array (**GSE96 **[106], **GSE6731 **[107] and **GSE1402 **[108]). 

A total of 81 transcriptional profiles encompassing cancer cell lines with nine histological origins and samples from six normal tissues were recovered.  Further details can be found in the legends of Tables 1 and 2. All datasets were generated by downloading and processing CEL files. They were preprocessed using Robust Multichip Average (RMA) [109,110] and then transformed from log_2_ values to linear scale values, and normalized per gene to the median value of its level of expression across 81 samples, as implemented in GeneSpring GX 7.3.1 (Silicon Genetics - ). The RMA preprocessing algorithm includes background and quantile normalization steps [109,111]. Although background correction, as first step analysis, has been computed separately for each array, all the other procedures performed by using RMA (normalization and summarization), have been performed across all the arrays (RMA is a multiple-array method). Normalization is necessary so that multiple chips can be compared to each other, and analyzed together. The normalization procedure is aimed at making the distributions identical across arrays. RMA usually gives very accurate normalizations. Note that, RMA implemented in GeneSpring GX 7.3.1, all the arrays are used and no chip is discarded. We used RMA analysis as compared to other tools of analysis, because, as described in several papers [112,113], it successfully reduces the variance of low abundance transcripts and better distinguishes differentially expressed transcripts from those that are unchanging, by using controlled datasets in which known quantities of specific mRNAs have been added to a common reference pool, [109,110,114]. 

### Transformation-dependent, transcriptional remodeling of the PKA pathway-encoding genes in 60 human cancer cell lines (NCI60) and 21 human normal tissues

We identified and gathered the transcriptional profile for 41 genes encoding proteins involved in the PKA pathway (adenylyl cyclases -ADCY-, phosphodiesterases -PDE-, A-kinase anchor proteins -AKAP-, cAMP-dependent transcriptional factors -TF-, PKA catalytic subunits -PRKAC- and PKA regulatory subunits -PRKACR-, Table 3).

In order to identify specific variations in the expression pattern of the selected PKA pathway-related genes both in normal and transformed samples, different tools of analysis were used.

Initially, the PKA pathway related genes expression profiles, observed in transformed samples as compared to normal samples, were evaluated by analysis of variance (ANOVA). Such statistical linear modeling procedure, that partitions the total variance into parts corresponding to various sources in the model [115,116] have been successfully used to analyze microarray data [117-120]. In order to model and test the hypothesis that the expression of genes of PKA pathway was different between normal tissues and transformed cell lines, the following comparisons were used: *Expression of gene_i_ (where i=i-esimo) of Normal Tissues vs. Transformed cell lines* (Figure 1), and a p-value < 0.05.

The same data-set was then analyzed through unsupervised hierarchical clustering [121] (as implemented in the GeneSpring platform). Two-way hierarchical clustering was performed on RMA-generated linear scale expression levels using the Pearson correlation coefficient as the measure of similarity and complete linkage clustering [122]. The results of this process are dendrograms, in which short branches connect very similar elements, and longer branches join elements with diminishing degrees of similarity. The vectors used were sample - normal tissues and transformed cells- and expression of genes of PKA pathway-related genes and the arms were classified by different variables:  *Conditions* and *Tissues*, (Figure 2). 

### Analysis of mutational status of the NCI60 cell lines and correlation with tissue-specific PKA pathway gene regulation

The 60 cell lines were sorted according to mutational status, using the information provided by Catalogue Of Somatic Mutations In Cancer () [123]. This database holds somatic mutation data and other information related to human cancer cell lines and tissues, and can be interrogated through a series of web pages to provide a graphical or tabular view of the data along with various export options. We could sort the NCI60 cell lines in 4 subgroups presenting mutational activation of genes encoding components of the Ras pathway, of the PI3K pathway, of other pathways or for which no information was available, (Table 5).

In order to identify specific variations in the expression pattern of the PKA pathway-related genes in these 4 subgroups, different tools of analysis were used.

We applied unsupervised Principal Component Analysis (PCA) [124,125] to establish the interrelationships among the samples used in our study. PCA is a statistical method that can be used to reduce complex data sets with multiple variables into significantly smaller numbers of variables (known as components), which retain the relevant variance information used to distinguish the sample groups from another. By visualizing projections of these components in low-dimensional spaces, we were able to observe the grouping of samples reflecting underlying patterns in their gene expression profiles. PCA on the mean centered and scaling data was used to model the effects of oncogene-dependent transformation on the gene expression. The following comparisons were performed: *Expression of gene_i_ of Normal Tissues vs. PI3K mutation cell lines; vs. Ras mutation cell lines; vs. Not Tested mutation cell lines; vs. Other Mutation cell lines.*

Also in this case, in order to model and test the hypothesis that the expression of genes of PKA pathway was different between normal tissues and the four subgroups previously identified, we applied one-way ANOVA by using the following comparisons:   *Expression of gene_i_ (where i=i-esimo) of Normal Tissues vs. PI3K mutation cell lines; vs. Ras mutation cell lines; vs. Not Tested mutation cell lines; vs. Other Mutation cell lines*, (Figure 3D), and a p-value < 0.05.

The data-set of 41 genes was then analyzed through unsupervised hierarchical clustering  (Pearson correlation coefficient and complete linkage clustering). The vectors used were sample - oncogene-dependent transformed cells - and expression of genes of PKA pathway-related genes. The results of this process are dendrograms, in which the arms were classified by different variables: *Tissue, mutation and Pathway* (Figure 4).

### Computational analysis of promoters of differentially regulated PKA pathway-encoding genes and identification of transcriptional factor binding sites

In order to identify Transcriptional Factor Binding Sites (TFBS) present in promoters of co-regulated genes, the 41 PKA pathway-encoding genes were sorted, relative to their level of expression, in three groups: Strong (>1), Average (=1) and Low (<1), where 1 is the expression value calculated by RMA. Each groups was identified in each sample group, i.e., Normal Tissues, cell lines carrying mutation(s) in Ras pathway-encoding genes, cell lines carrying mutation(s) in PI3K pathway-encoding genes, cell lines carrying mutation(s) in other pathways, cell lines Not Tested for mutation, thus generating 15 subgroups. A TFBS was called present only when present in more than 70 % of promoters within each group. 

Proximal promoter regions - defined as 500 nt upstream and 100 nt downstream from the transcription start site (TSS), automatically assigned to genes on the basis of 5' cap-site databases integrated into promoter identification program - were identified using Eldorado (gems launcher, Genomatix [126]) and the Genomatix Promoter Database. 

TFBS in the promoter regions were identified by using ModelInspector and the Genomatix Promoter Database, comprising a total of 519 matrices from 154 families (Matrix Family Library, on Vertebrates, Version 7.1, June 2008). The Matrix Family Library is based on 260,000 human, mouse, and rat promoter sequences, with an average length of 650bp. Analysis on the 41 PKA pathway-encoding genes was performed with a threshold of 1.0 for the core similarity -that is reached only when the highest conserved bases of a matrix match exactly in the sequence- and a value of 0.85 for the Optimized matrix threshold [127]. Optimized matrix threshold is the optimized value defined in a way that a minimum number of matches is found in non-regulatory test sequences. This value, when is higher than 0.80, permits the reduction of false positive matches.  

The total number and frequency (i.e., the ratio between the total number of TBFS and the number of promoters present within each subgroup) of each TFBS within each subgroup were calculated. The frequency of each TFBS called present in each of the 15 subgroups of PKA pathway-encoding genes was compared with the frequency of the same TFBS within the Matrix Family Library on Vertebrates. TFBS enrichment was scored based on p-value generated by hypergeometric distribution and calculated with the 2-tailed Fisher's exact test, implemented through the use of a 2 x 2 contingency table (Figure 5).

In order to identify differences between the 15 groups a two-way hierarchical clustering (by using as vectors sample and TFBS) was applied by using the total number values and the frequency values of each TFBS identified in ≥ 70% of the promoters in each group. The total number value was transformed in the log_2_ and used in the hierarchical clustering by using the Pearson correlation coefficient as the measure of similarity and complete linkage clustering  (Figure 6).

### Promoter data mining

To identify known transcription factor binding sites in the promoter sequences of PKA pathway-encoding genes, the annotated promoter and associated information have been retrieved from Transcriptional Regulatory Element Database (TRED) [128] () and from NCBI (). Both web sites are freely accessible. The results have been shown in the Table 6.

## Competing interests

The authors declare that they have no competing interests.

## Authors' contributions

CB, MV and FC conceived the experiments and wrote the manuscript. CB and FC carried out the data analysis. LA, MV and FC participated in data analysis. All authors read, edit and approved the final manuscript.
